# A *tert*-Butyldiphenylsilyl-Containing Polyimide-Based Chemosensor for Sequential Detection of Fluoride Ions and Trace Water in Organic Solvents

**DOI:** 10.3390/molecules28247987

**Published:** 2023-12-07

**Authors:** Yancheng Wu, Manyu Lian, Guotao Huang, Yangfan Zhang, Ningbo Yi, Liyong Tian, Feng Gan, Chunping Ma

**Affiliations:** School of Textile Materials and Engineering, Wuyi University, Jiangmen 529020, Chinayiningbo@wyu.edu.cn (N.Y.); gf@dhu.edu.cn (F.G.);

**Keywords:** *tert*-butyldiphenylsilyl-containing polyimide, colorimetric and ratiometric chemosensor, fluoride ions, trace water

## Abstract

A *tert*-butyldiphenylsilyl-containing polyimide (**PI-OSi**) has been established as a colorimetric and ratiometric chemosensor for rapid detecting fluoride ions (F^−^). The UV-vis absorbance ratio value (A_322_/A_288_) of **PI-OSi** in a DMF solution displays a wide linear range change to F^−^ concentrations with a detection limit (DL) value of 2.13 μM. Additionally, adding incremental amounts of F^−^ to a DMF solution of **PI-OSi** shows an immediate color change to yellow and finally to green from colorless. More interestingly, the resulting **PI-OSi** plus F^−^ system (**PI-OSi**·F) could detect trace water in DMF. The A_292_/A_322_ value of **PI-OSi**·F almost linearly increases with low water content, which suggests convenient quantitative sensing of trace water content in DMF. The DL value of **PI-OSi**·F for sensing water in DMF is determined to be 0.00149% (*v*/*v*). The solution color of **PI-OSi**·F returns to colorless when the water content increases, indicating that **PI-OSi**·F can conveniently estimate water content in DMF by naked-eye detection. The detection mechanisms confirmed by an ^1^H NMR study and a DFT calculation involve a F^−^-induced desilylation reaction of **PI-OSi** to form phenolate anion followed by protonation with trace water. Finally, **PI-OSi** film was fabricated for the colorimetric detection of F^−^ and water in CH_3_CN.

## 1. Introduction

As an essential micronutrient, fluoride ions (F^−^) can effectively avoid dental caries and contribute to the treatment of osteoporosis in the right amount [1]. However, excess F^−^ exposure may damage human health and destroy the ecological environment to a certain extent [2,3]. For these reasons, the design of a simple and accurate F^−^ chemosensor has become the focus of current attention, and its technology is becoming more and more mature [4,5,6,7,8,9]. At present, colorimetric chemosensors with color change detected by the naked-eye are effectively applied to detect F^−^ [10,11,12,13,14]. Nevertheless, many colorimetric F^−^ chemosensors are based on the sensing mechanism of hydrogen bonding but usually show poor selectivity because they are susceptible to interference by basic anions such as CN^−^, AcO^−^, and H_2_PO_4_^−^ [15,16,17,18]. Researchers have found that F^−^ has a high affinity for silicon (Si), so F^−^ and Si atoms can form stable Si-F bonds. Based on this reactive mechanism, reactive chemosensors for sensing F^−^ with high selectivity can be designed [19,20,21]. In addition, compared with many small organic molecule-based reactive F^−^ chemosensors, polymer-based reactive F^−^ chemosensors have some additional advantages, such as good photo-, thermo-, and chemical stability [22,23]. However, due to the relatively slow reaction rate, reactive polymeric F^−^ chemosensors often need excessive F^−^ and have a long response time [24]. Moreover, reactive F^−^ chemosensors often can only be used for the one-off detection of F^−^ due to their irreversibility [19,21]. It is very meaningful if a reactive F^−^ chemosensor after use to detect F^−^ is able to continue to detect other analytes [20].

Water is very important to our daily life, but water molecules often exist as impurities in organic solvents, so it is necessary to detect trace water in chemical reactions and industry [25,26]. Due to the advantages of naked-eye detection, high sensitivity, quick response, and convenience to use in situ, colorimetric water chemosensors are often prepared to detect water [27,28,29,30,31,32,33]. However, most colorimetric chemosensors for sensing water are based on small organic molecules, which often show poor photostability [33,34,35,36] and difficulty in sensing trace amounts of water [29]. Recently, it has been found that polymer-based water chemosensors can effectively improve photostability and sensitivity [36,37]. For example, Wang et al. [38] prepared an aggregation-induced emission (AIE) polymer containing diketopyrrolopyrrole and triphenylamine as a fluorescence sensor for sensing trace water in THF with a detection limit (DL) value of 0.005% (*v*/*v*). Zhao et al. [39] developed a ratiometric and colorimetric sensor based on hydroxyl-containing polyimide–fluoride complexes, which show high sensitivity for water detection with extremely low DL values of 0.00084% and 0.0015% in DMSO and DMF. Therefore, it is meaningful to design polymer-based water chemosensors with high sensitivity.

**Scheme 1:**
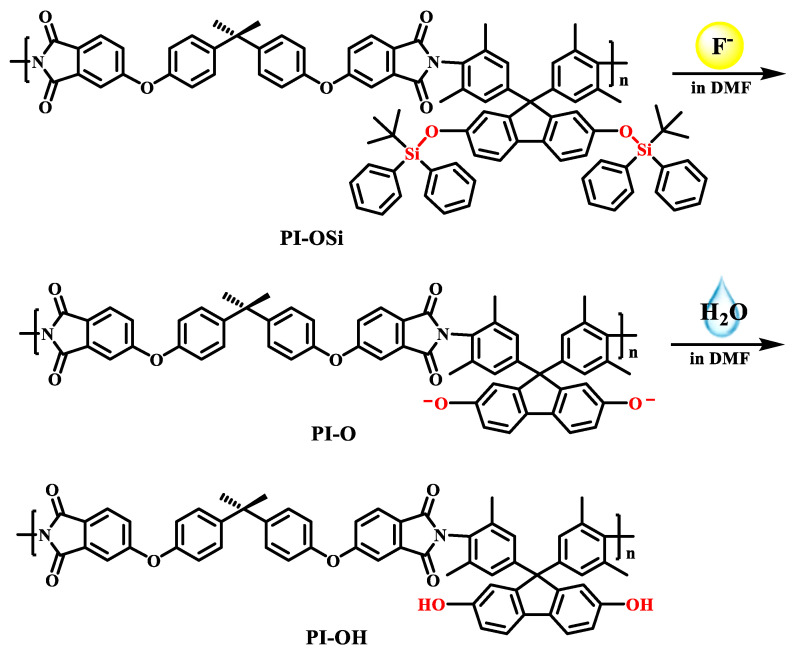
The structures and sensing mechanisms of **PI-OSi** for F^−^ and **PI-OSi**·F for trace water in DMF.

In this work, a *tert*-butyldiphenylsilyl-containing polyimide (namely, **PI-OSi**) was easily synthesized by a simple and efficient silicon etherification reaction of a hydroxyl-containing polyimide (namely **PI-OH**) with a *tert*-butyldiphenylsilyl chloride (**TBDPSCl**). The as-obtained **PI-OSi** contains an O-Si bond, which possesses a strong nucleophilic interaction with F^−^. As expected, **PI-OSi** is potentially designed as a reactive chemosensor for the detection of F^−^ with high selectivity in which the specific fluoride-induced desilylation reaction led to the formation of a phenolate anion (Scheme 1). Thus, the intramolecular charge transfer (ICT) in the resulting polymer chains is obviously increased and can be analyzed by observing the changes in some photophysical properties, e.g., UV-vis absorption and fluorescence spectra. Another interesting phenomenon is that the resulting **PI-OSi** plus F^−^ system (**PI-OSi**·F) could sensitively detect trace water in a DMF solvent because the trace water is able to protonate the phenolate anion (Scheme 1). The F^−^ recognition behavior of the chemosensor **PI-OSi** and the water sensing of chemosensor **PI-OSi**·F were observed and examined by UV-visible spectroscopy and naked-eye detection.

## 2. Results and Discussion

### 2.1. Synthesis and Characterization of **PI-OH** and **PI-OSi**

The hydroxyl-containing polyimide (**PI-OH**) was prepared via a one-step polymerization of a diamine monomer, DMAFH, and a commercially available dianhydride, **BPADA,** in an NMP solution. The *tert*-butyldiphenylsilyl-containing polyimide (**PI-OSi**) was prepared via a simple and efficient silicon etherification reaction of **PI-OH** and **TBDPSCl** at room temperature. The chemical structures of PI-OH and PI-OSi are well characterized by ^1^H NMR (for the corresponding spectra, see Figure S1 in the Supporting Information (SI)).

### 2.2. Spectral Sensing of F^−^ by **PI-OSi**

**Figure 1:**
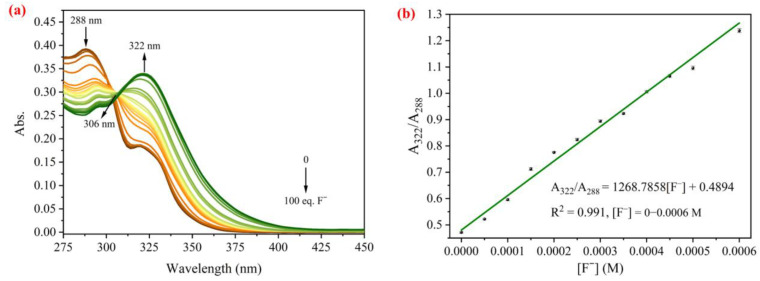
(**a**) UV-*vis* absorption spectra of **PI-OSi** (10 µM in anhydrous DMF) during titration with F^−^ (0–100 eq.); (**b**) A_322_/A_288_ ratio of **PI-OSi** as a function of the F^−^ concentration in DMF solution.

**PI-OSi** contains an O-Si bond of O-*tert*-butyldiphenylsilyl (O-TBDPS), which possesses a strong nucleophilic interaction with F^−^. Therefore, **PI-OSi** is potentially designed as a reactive chemosensor for the highly selective detection of F^−^, in which the fluoride-induced desilylation reaction could be analyzed by observing the changes in the spectral properties and solution color. To test this, the UV-*vis* absorption spectrum measurement was examined first by adding incremental amounts of F^−^ to an anhydrous DMF solution of **PI-OSi** (Figure 1). The UV-*vis* absorption spectrum of the free **PI-OSi** displayed a prominent absorption peak at 288 nm, accompanying a weak shoulder peak at around 322 nm. Adding the incremental amounts of F^−^ to the DMF solution of **PI-OSi** results in a gradual diminishment in the intensity of the absorption peak at 288 nm, accompanying a gradual increment in the intensity of the absorption peak at 322 nm. And, an isosbestic point at 306 nm was observed, indicating that one stable species at least is present at equilibrium [40]. These results originated from the F^−^-induced desilylation reaction of O-TBDPS in polymer chains to form the phenolate anion, which shows increased electron density compared with O-TBDPS side groups. Therefore, the ICT in the resulting polymer backbone is strongly increased, resulting in a distinct red shift in the UV-*vis* absorption spectrum.

The functional relationship of A_322_/A_288_ values of **PI-OSi** with F^−^ concentration is shown in Figure 1b. The A_322_/A_288_ value change with F^−^ concentration (0–0.6 mM) exhibits a good linear relationship (R^2^ = 0.991). The DL value of **PI-OSi** for sensing F^−^ is calculated to be 2.13 µM. These results indicate that chemosensor **PI-OSi** is capable of serving as a ratiometric chemosensor for the highly sensitive detection of F^−^, which can be applied to the quantitative analysis of F^−^ concentrations in a DMF solution.

**Figure 2:**
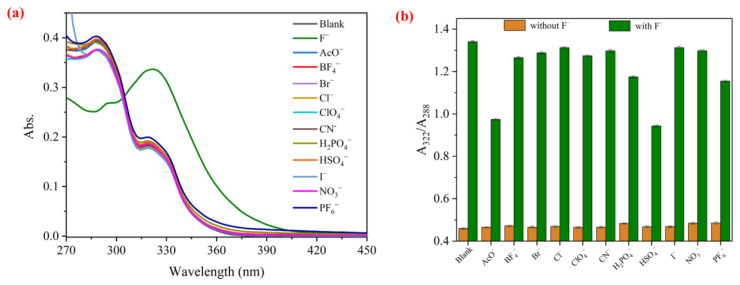
(**a**) UV-vis absorption spectra of **PI-OSi** (10 µM in anhydrous DMF) with various anions (100 eq.); (**b**) A_322_/A_288_ value of **PI-OSi** upon adding 100 eq. anions without and with F^−^.

Subsequently, the selectivity of sensor **PI-OSi** toward 12 different anions (100 eq.), i.e., F^−^, AcO^−^, BF_4_^−^, Br^−^, Cl^−^, ClO_4_^−^, CN^−^, H_2_PO_4_^−^, HSO_4_^−^, I^−^, NO_3_^−^, PF_6_^−^, and 5 metal cations (100 eq.), i.e., Na^+^, Al^3+^, Ba^2+^, Co^2+^, and Cs^+^, was investigated using the UV-*vis* absorption spectrum. It shows that no significant UV-*vis* absorption spectra changes were observed with the addition of these anions, except F^−^ (Figure 2a). When 100 eq. F^−^ was added to the DMF solution of chemosensor **PI-OSi**, a distinct red shift in the resulting UV-*vis* absorption spectrum was immediately observed. In addition, adding the abovementioned metal cations into the DMF solution of chemosensor **PI-OSi** could not cause any change in UV-*vis* absorption spectra (Figure S2 in SI). Furthermore, interference experiments were conducted in the presence of competitive anions. Adding F^−^ to the solution of chemosensor **PI-OSi** in the presence of other anions led to a similar trend in UV-*vis* absorption spectra to that with the addition of F^−^ only (Figure S3 in SI). As shown in Figure S3 in SI, adding F^−^ to the solution of chemosensor **PI-OSi** in the presence of other anions also caused an obvious red shift in the resulting UV-vis absorption spectrum. Figure 2b shows A_322_/A_288_ value changes after adding 100 eq. anions without and with F^−^. Obviously, the UV-*vis* absorption spectra response of chemosensor **PI-OSi** toward F^−^ was not affected by other anions [40,41].

### 2.3. Visual Sensing of F^−^ by **PI-OSi**

**Figure 3:**
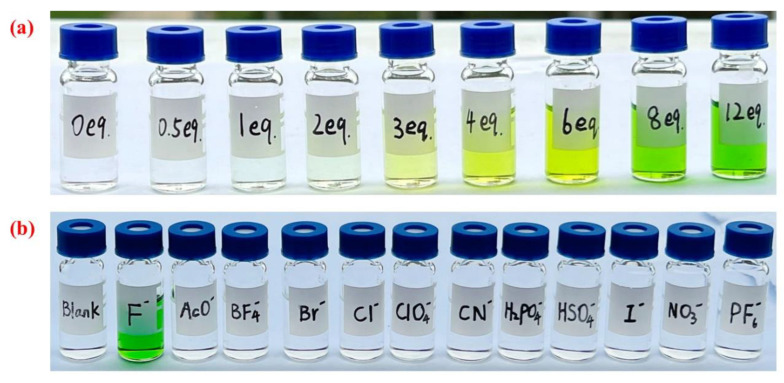
The color changes in chemosensor **PI-OSi** (1 mM in anhydrous DMF) upon adding (**a**) various concentrations of F^−^ (0–12 eq.) and (**b**) various anions (12 eq.).

Visual sensing can be implemented without a precise instrument, which is more important for practical application. The sensing behavior of chemosensor **PI-OSi** for the sensitive and selective detection of F^−^ was further investigated by naked-eye detection. While adding incremental amounts of F^−^, chemosensor **PI-OSi** (1 mM in DMF) progressively changed to yellow and finally to green from colorless (Figure 3a). This visible color change occurred almost immediately after adding F^−^ to the DMF solution of chemosensor **PI-OSi**. Adding 1 eq. F^−^ into the DMF solution of chemosensor **PI-OSi** caused an obvious color change, and the color change degree of the DMF solution of chemosensor **PI-OSi** varied with increasing F^−^ concentration. These results indicate that the chemosensor **PI-OSi** can be conveniently used for the practical estimation of F^−^ concentration by naked-eye detection. Fortunately, adding other anions to the DMF solution of chemosensor **PI-OSi** does not cause a color change (Figure 3b). The results indicate that **PI-OSi** is able to serve as a “naked-eye” chemosensor for the highly sensitive and selective detection of F^−^ with a fast response time.

The distinct UV-*vis* absorption spectrum and color changes in the DMF solution of chemosensor **PI-OSi** for F^−^ originated from a specific fluoride-induced desilylation reaction of O-TBDPS in polymer chains to form the phenolic hydroxyl anion. The as-formed phenolic hydroxyl anion, once encountering trace water, could easily combine with protons to form **PI-OH** (Scheme 1), which led to the corresponding changes in the UV-*vis* absorption spectrum and the solution color. Therefore, the **PI-OSi** plus F^−^ system (**PI-OSi**·F) may be able to be used as a chemosensor for sensing trace water in DMF. In our case, the DMF solution of chemosensor **PI-OSi**·F with the trace water caused an obvious blue shift in the UV-*vis* absorption spectra (Figure S4 in SI) and a color change (Figure S5 in SI). In addition, adding F^−^ into other organic solutions (such as THF, DMAc, and 1,4-dioxane) of chemosensor **PI-OSi** caused an obvious red shift in the UV-*vis* absorption spectrum (Figure S6 in SI). And, subsequently, adding trace water into the abovementioned solution of the **PI-OSi** plus F^−^ system led to a blue shift in the resulting UV-*vis* absorption spectrum (Figure S6 in SI). Therefore, these results suggest that **PI-OSi** is able to sequentially detect fluoride ions and trace water in water-soluble solvents, which can dissolve **PI-OSi**.

### 2.4. Spectral Sensing of Water by **PI-OSi**·F 

**Figure 4:**
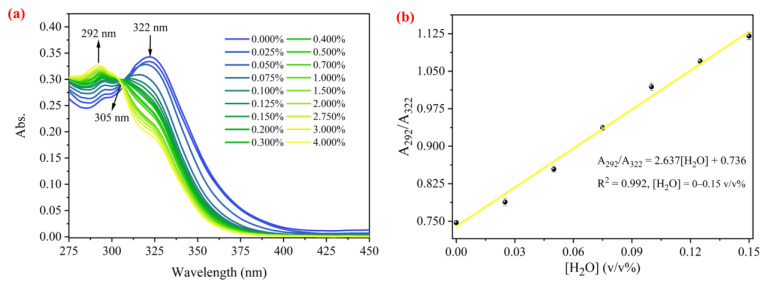
(**a**) UV-*vis* absorption spectra of **PI-OSi**·F (10 µM in DMF + 50 eq. F^−^) during titration with water (0–4.000%, *v*/*v*); (**b**) A_292_/A_322_ ratio of **PI-OSi**·F as a function of water content in DMF solution.

The change in the UV-vis absorption spectra of **PI-OSi**·F with the addition of increasing amounts of water in DMF was systematically investigated (Figure 4). As the water content increased, the absorption peak at 322 nm decreased gradually, accompanying a gradual increase in the absorption peak at 292 nm (Figure 4a). The A_292_/A_322_ values show a good linear relationship (R^2^ = 0.992) with water content in DMF up to 0.15% (*v*/*v*), implying that **PI-OSi**·F could be used to detect water quantitatively at a trace level (Figure 4b). On the basis of UV-vis absorption titration data, the DL value of chemosensor **PI-OSi**·F for sensing water in DMF was determined to be 0.00149% (*v*/*v*).

### 2.5. Visual Sensing of Water by **PI-OSi**·F

**Figure 5:**
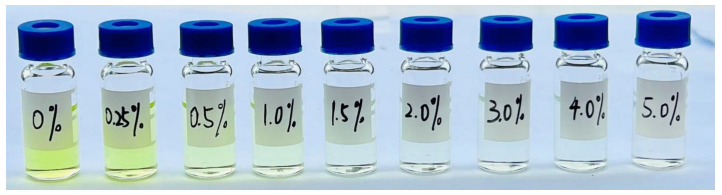
The solution color change in chemosensor **PI-OSi**·F (1 mM **PI-OSi** in DMF + 6 eq. F^−^) with different water fractions.

Changes in the water content in the DMF solution of **PI-OSi**·F (1 mM **PI-OSi** + 6 eq. F^−^) also triggered significant variational color change (Figure 5). When the water content increased from 0% to 5.0% (*v*/*v*), the solution color of chemosensor **PI-OSi**·F gradually changed from light yellow-green to colorless, and the solution color change was instantaneous. Similarly, the color change degree of the DMF solution of chemosensor **PI-OSi**·F varied with higher water content, indicating that chemosensor **PI-OSi**·F can be conveniently used for the practical estimation of water content by naked-eye detection. These results might pave the way for building a colorimetric chemosensor for the real-time detection of trace water in DMF with high sensitivity.

### 2.6. Reaction Mechanism

**Figure 6:**
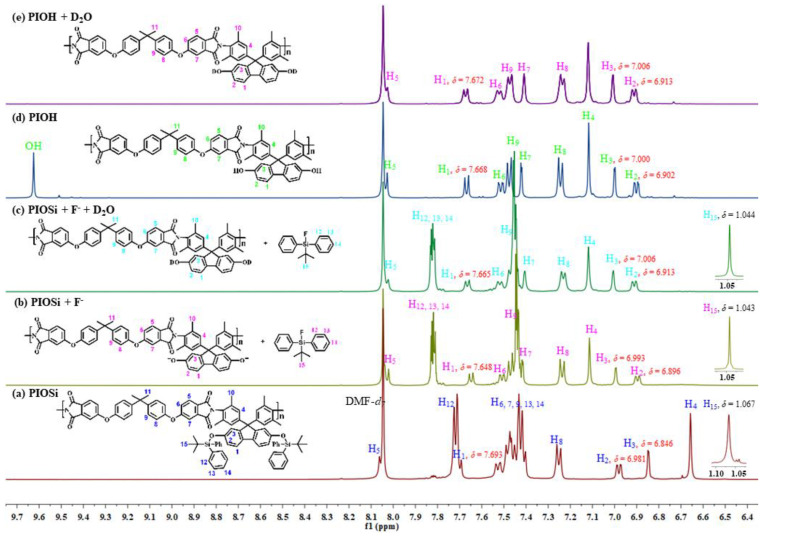
Partial ^1^H NMR spectra of (**a**) **PI-OSi** (10 mM); (**b**) **PI-OSi** (10 mM) + 0.2 eq. F^−^; (**c**) **PI-OSi** (10 mM) + 0.2 eq. F^−^ + 4 *v*/*v*% D_2_O; (**d**) **PI-OH** (10 mM); and (**e**) **PI-OH** (10 mM) + 4 *v*/*v*% D_2_O.

To gain insight into the possible sensing mechanisms shown in Scheme 1, the ^1^H NMR experiments were first studied, and the chemical shift values of key protons are listed in Table 1. At first, when 0.2 eq. F^−^ was added to **PI-OSi** (0.5 mL, 10 mM in DMF-*d*_7_), the signal peaks belonging to the Si-C(CH_3_)_3_ (H_l5_, see inset in Figure 6a and Table 1) of **PI-OSi** at 1.067 ppm moved to the upfield region (1.043 ppm, H_15_, see inset in Figure 6b and Table 1). Moreover, a multiple peak at 7.843–7.799 ppm attributed to Ar-H of *tert*-butyldiphenylfluorosilane (H_12,13,14_, Figure 6b) appeared. Compared with the ^1^H NMR spectrum of pure **PI-OH** (Figure 6d), the chemical shift values of aromatic protons in the **PI-OSI** with F^−^ system are almost the same except for the aromatic protons H_1,2,3_ being adjacent to O-TBDPS. The signal peaks of H_1,2,3_ shifted to the upfield region (Figure 6b and Table 1). This phenomenon confirms the F^−^-induced desilylation reaction of O-TBDPS in polymer chains to form the phenolic hydroxyl anion, causing an increase in electron density compared with **PI-OH**. After adding trace heavy water to the **PI-OSi** with F^−^ system, the signal peaks of H_1,2,3_ shifted downfield (Figure 6c and Table 1). Although there were still little differences with those of pure **PI-OH**, the signal peaks of H_2,3_ were consistent with the ^1^H NMR spectrum of **PI-OH** with heavy water (Figure 6e and Table 1). This suggests that the phenolic hydroxyl anion was protonated to the generation of **PI-OH** in the presence of trace water. The signal peak of the hydroxyl group in the ^1^H NMR spectrum of **PI-OSi**·F with water (Figure 6c) did not appear because of the rapid chemical exchange with D_2_O, which agrees with the ^1^H NMR spectrum of pure **PI-OH** with D_2_O (Figure 6e).

**Figure 7:**
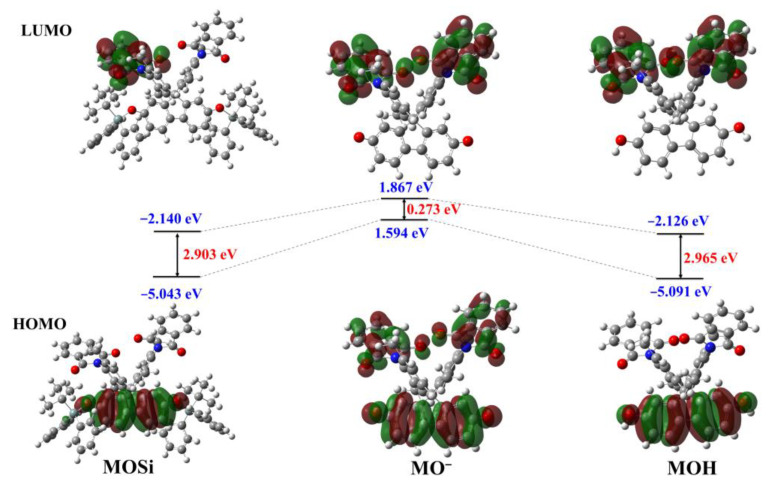
The frontier molecular orbitals of **MOSi**, **MO**^−^, and **MOH**.

The above ^1^H NMR experiments can be explained by the chemical structure of the sensor systems changing when the F^−^ and subsequent water were added. To further understand the mechanism of the UV-*vis* absorption spectra response, a DFT calculation optimized at the B3LYP/6-31G theoretical level was performed. The simplified unit structures of polymers as the model compounds were used to optimize structure geometries (Figure S7 in SI). The calculated energy of the highest occupied molecular orbital (E_HOMO_) and the lowest unoccupied molecular orbital (E_LUMO_) and their energy gaps (ΔE) of **MOSi**, **MO^−^**, and **MOH** are shown in Figure 7. The E_HOMO_ value of **MO**^−^ (1.594 eV) is much higher than that of **MOSi** (−5.043 eV), indicating that the fluoride-induced desilylation reaction of O-TBDPS in polymer chains that forms the phenolic hydroxyl anion enhances the electron-donating ability of diamine fragments. In addition, the ΔE values of **MO^−^** (0.273 eV) are lower than those of **MOSi** (2.903 eV), meaning that the ICT in **MO^−^** is significantly increased compared with that in **MOSi**. This phenomenon can explain how adding F^−^ to **PI-OSi** led to the red shift of the resulting UV-*vis* absorption spectrum. For **MOH**, the E_HOMO_ and ΔE values are about the same as those for **MOSi**, indicating that the degree of ICT in **MOH** is almost equal to that in **MOSi** and much lower than in **MO^−^**. This phenomenon can explain the UV-*vis* absorption spectrum of chemosensor **PI-OSi**·F after the addition of water being blue shifted to a similar state to that of **PI-OSi**.

### 2.7. Portable Test Polyimide Film-Based Chemosensor Device

**Figure 8:**
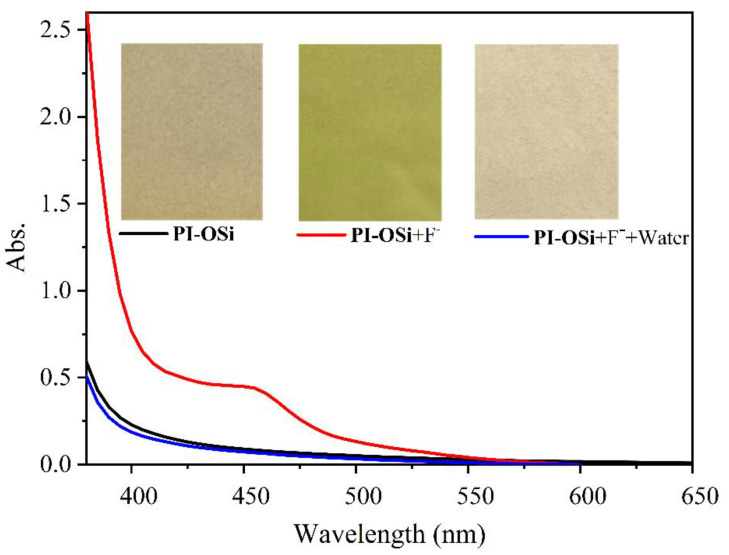
UV-*vis* absorption spectral changes in **PI-OSi** film upon immersion in dry and wet CH_3_CN solutions containing 10^−2^ M of F^−^. (Inset: color changes in **PI-OSi** film).

Polymeric film materials can directly act as a portable chemosensor device based on colorimetric detection with many advantages, such as its handy, durable, easily operated, and eco-friendly nature [39,40]. Polyimides (PIs) possess superior film-forming performance. PI films, with their excellent integrative properties, are effectively employed even in extreme conditions. Therefore, PI materials are excellent candidates for the design and fabrication of polymer film-based chemosensor devices. **PI-OSi** film is insoluble in some water-miscible solvents, such as CH_3_CN, which provides potential for using **PI-OSi** films to achieve F^−^ and water detection in CH_3_CN. Thus, the possibilities of using a **PI-OSi** film-based chemosensor device for detecting F^−^ and water in CH_3_CN were explored. As shown in Figure 8, the UV-vis absorbance intensity of **PI-OSi** film after immersion in a dry CH_3_CN solution containing 10^−2^ M of F^−^ was dramatically increased at visible wavelengths. And, the color of **PI-OSi** film changed accordingly to yellow-green from pale yellow (see inset in Figure 8). However, almost no UV-vis absorbance intensity or color changes were observed after immersing **PI-OSi** film into a wet CH_3_CN solution containing 10^−2^ M of F^−^. These results suggest that water can affect the interaction between **PI-OSi** film and F^−^. Therefore, a colorimetric detection of F^−^ and water in CH_3_CN by **PI-OSi** film may be achievable.

## 3. Experimental Setup

### 3.1. Reagents and Apparatus

The diamine monomer **DMAFH** was prepared in our laboratory according to the literature [42]. 4,4′-(4,4′-isopropylidenediphenoxy)diphthalic anhydride (**BPADA**, 98%), from TCI Tokyo Chemical Industry Co., Ltd. (Tokyo, Japan), was dried in a vacuum oven at 140 °C. *tert*-Butylchlorodiphenylsilane (**TBDPSCl**) and imidazole were purchased from Energy Chemical Co., Ltd. (Anhui, China). The testing anions (including F^−^, AcO^−^, BF_4_^−^, Br^−^, Cl^−^, ClO_4_^−^, CN^−^, H_2_PO_4_^−^, HSO_4_^−^, I^−^, NO_3_^−^, and PF_6_^−^) used as the tetrabutylammonium (TBA^+^) salts, except for cyanide ions, and the extra-dry solvents (including DMF, THF, DMAc, and 1,4-dioxane, >99.9%, water ≤ 30 ppm) used as 0% (*v*/*v*) water for the water-sensing measurements were obtained from Energy Chemical Co., Ltd. Tetrabutylammonium cyanide was purchased from Sigma-Aldrich LLC (Burlington, MA, USA). The testing metal ions (including Na^+^, Al^3+^, Ba^2+^, Co^2+^, and Cs^+^) used as chloride were purchased from Aladdin. Deionized water was prepared in our laboratory with a water purification system (UPT-II-20T). The other chemical reagents were commercially available and were used without further purification.

The UV-*vis* absorption experiments were measured on a Shimadzu UV-2700 spectrophotometer (Kyoto, Japan). ^1^H-NMR experiments were carried out on a Bruker Avance DRX500 NMR Spectrometer in DMF-*d*_7_ (Billerica, MA, USA), as the solvent and its residual peak were used as an internal reference.

### 3.2. General Procedure for UV-vis Absorption Spectra Measurements

**PI-OSi** was dissolved in extra-dry DMF to acquire 1 mM (calculated in terms of the monomeric units) stock solution, which was further diluted to 10 μM for the F^−^ sensing measurements. The solutions of TBA^+^ salts (1 mM) were prepared by dissolving them in extra-dry DMF.

Deionized water was utilized for the water titration study. The increasing water content was added to the solutions of the **PI-OSi** plus F^−^ system in extra-dry DMF to create the DMF aqueous solutions with the required water content.

All of the above test solutions (2 mL) were placed in a quartz cell for the UV-vis absorption spectra measurements.

### 3.3. Calculation Method for Detection Limit (DL)

On the basis of UV-vis absorption titration data, the DL value was calculated by using the formula DL = 3σ/*k* [43]. Herein, σ is the standard deviation of a blank, and *k* is the slope of the calibration line.

### 3.4. Synthesis

#### 3.4.1. Synthesis of **PI-OH**

As shown in Scheme S1, the diamine monomer **DMAFH** (1.746 g, 4 mmol), **BPADA** (2.082 g, 4 mmol), and 20 mL of NMP were added to a 250 mL round-bottom flask and stirred at room temperature for 12 h under the protection of N_2_. Then, the reaction temperature was raised successively to 120 °C for 1 h and 180 °C for 3 h to achieve complete imidization. After the reaction, **PI-OH** resin was isolated by precipitation into ethanol. Finally, the crude products dissolved in DMAc were reprecipitated into ethanol to give pure **PI-OH** resin.

**PI-OH**: ^1^H NMR (500 MHz, DMF-*d*_7_) δ = 9.62 (s, 2H), 8.03 (s, 2H), 7.67 (d, *J* = 8.2 Hz, 2H), 7.52 (d, *J* = 8.2 Hz, 2H), 7.48 (d, *J* = 8.7 Hz, 4H), 7.42 (d, *J* = 2.1 Hz, 2H), 7.24 (d, *J* = 8.6 Hz, 4H), 7.12 (s, 4H), 7.00 (s, 2H), 6.90 (d, *J* = 10.0 Hz, 2H), 2.09 (s, 12H), 1.77 (s, 6H).

#### 3.4.2. Synthesis of **PI-OSi**

A round-bottom flask was charged with **PI-OH** resin (1.842 g; 2 mmol), TBDPSCl (0.904 g; 6 mmol), and imidazole (0.408 g; 6 mmol) in 20 mL of DMF. The reaction mixture was stirred at room temperature under N_2_ for 24 h. After the reaction, the homogeneous solution was slowly poured into stirred ethanol (400 mL) to afford a fibrous precipitate, which was washed thoroughly with ethanol and dried in a vacuum oven at 80 °C. Finally, the crude **PI-OSi** precipitate was reprecipitated by CHCl_3_-ethanol to give pure **PI-OSi** precipitate.

**PI-OSi**: ^1^H NMR (500 MHz, DMF-*d*_7_) *δ* = 8.06 (s, 2H), 7.77–7.66 (m, 10H), 7.54–7.39 (m, 20H), 7.25 (d, *J* = 8.5 Hz, 4H), 6.98 (d, *J* = 6.7 Hz, 2H), 6.85 (s, 2H), 6.66 (s, 4H), 1.93 (s, 12H), 1.77 (s, 6H), 1.07 (s, 18H).

### 3.5. Preparation of **PI-OSi** Film

The DMAc solution was made by dissolving **PI-OSi** with the solid content of 15 wt. %. The abovementioned polymer solution was poured into the glass substrate, which was heated in vacuo at 50 °C for 12 h to remove most of the solvent; then, the semi-dried film was further dried at 100 °C for 3 h, at 150 °C for 3 h, and at 200 °C for 2 h to thoroughly remove the residual DMAc solvent. The obtained **PI-OSi** film was about 50 μm thick.

## 4. Conclusions

In this work, we have conveniently synthesized a *tert*-butyldiphenylsilyl-containing polyimide (**PI-OSi**) as a colorimetric and ratiometric chemosensor for rapid and highly sensitive and selective detection of F^−^ by employing fluoride-induced cleavage of O-Si bonds. The chemosensor **PI-OSi** shows a highly selective response toward F^−^ over other tested anions via a red shift of the UV-*vis* absorption spectrum and solution color changes in DMF with a detection limit (DL) value of 2.13 μM. Intriguingly, the prepared **PI-OSi** plus F^−^ system (**PI-OSi**·F) effectively detected the water content in the DMF solution, exhibiting a blue shift of the UV-*vis* absorption spectrum and a solution color change. The DL value of chemosensor **PI-OSi**·F for sensing trace water in DMF is as low as 0.00149% (*v*/*v*). The water-sensing mechanism was the F^−^-induced desilylation reaction of **PI-OSi** that formed the phenolic hydroxyl anion being protonated to generate **PI-OH** in the presence of trace water. In addition, **PI-OSi** film is directly used to visually detect F^−^ and water in CH_3_CN, which can serve as a handy polymer film-based chemosensor device for the detection of F^−^ and water in CH_3_CN. This work provides a novel design strategy to construct a polymer-based chemosensor for the rapid, highly selective and sensitive, and visible detection of F^−^ as well as trace water.

## Figures and Tables

**Table 1:** Summary of the chemical shift values of key protons ^a^.

Protons	PI-Osi (*δ*, ppm)	PI-OSi + F^−^ (*δ*, ppm)	PI-OSi + F^−^ + D_2_O (*δ*, ppm)	PI-OH (*δ*, ppm)	PI-OH + D_2_O (*δ*, ppm)
Ar-H_1_	7.693	7.648	7.665	7.668	7.672
Ar-H_2_	6.981	6.896	6.913	6.902	6.913
Ar-H_3_	6.846	6.993	7.006	7.000	7.006
SiC-CH_15_	1.067	1.043	1.044	--	--

^a^ All spectra were acquired on a 600 MHz NMR; solvent: DMF-*d*_7._

## Data Availability

Data are contained within the article and Supplementary Materials.

## Supplementary Materials

The following supporting information can be downloaded at https://www.mdpi.com/article/10.3390/molecules28247987/s1. Scheme S1: Synthesis of hydroxyl-containing **PI-OH** and tert-butyldiphenylsilyl-containing **PI-OSi**; Figure S1: ^1^H NMR spectra of (a) **PI-OH** and (b) **PI-OSi**; Figure S2: A322/A288 value of **PI-OSi** upon adding 100 eq. F- and five cations; Figure S3: UV-vis absorption spectra of **PI-OSi** upon the addition of 100 eq. F^-^ in the presence of 100 eq. different anions; Figure S4. Change in UV-vis absorption spectra for dry **PI-OSi** solutions (10 μM in DMF) after adding F^−^ and subsequent adding trace water; Figure S5: Changes in color of **PI-OSi** solutions (1 mM in DMF) with the addition of F^−^ (6 eq.) and subsequent addition of trace water (5%, *v*/*v*); Figure S6: Change in UV-vis absorption spectra for dry **PI-OSi** solutions (10 μM in (a) 1,4-Dioxane, (b) THF, (c) DMAc) after adding F^-^ and subsequent adding trace water; Figure S7: Structure of model compounds for theoretical calculation.
