# Supplementary material for: A tert-Butyldiphenylsilyl-Containing Polyimide-Based Chemosensor for Sequential Detection of Fluoride Ions and Trace Water in Organic Solvents
Source: Molecules. 2023 Dec 7;28(24):7987. doi: 10.3390/molecules28247987 (PMC10745802; doi:10.3390/molecules28247987)
Supplement: Supplementary file 1 [file molecules-28-07987-s001.zip › molecules-2687865-supplementary.pdf]

Supporting Information

A *tert*-Butyldiphenylsilyl-Containing Polyimide-Based Chemosensor for Sequential Detection of Fluoride Ions and Trace Water in Organic Solvents

Yancheng Wu *, Manyu Lian, Guotao Huang, Yangfan Zhang, Ningbo Yi, Liyong Tian, Feng Gan and Chunping Ma

School of Textile Materials and Engineering, Wuyi University, Jiangmen 529020, China; 15362253952@163.com (M.L.); 15029891361@163.com (G.H.); zyf@wyu.edu.cn (Y.Z.); yiningbo@wyu.edu.cn (N.Y.); tlydbd@163.com (L.T.); gf@dhu.edu.cn (F.G.); machunpingaa@126.com (C.M.)

* Correspondence: wyc@wyu.edu.cn

Contents

1. Synthesis and characterization of **PI-OH** and **PI-OSi**. (**Scheme S1** and **Figure S1**)..........................S2
2. A_{322}/A_{288} value of **PI-OSi** upon adding 100 eq. F^- and five metal cations. (**Figure S2**)..S3
3. UV-vis absorption spectra of **PI-OSi** upon the addition of 100 eq. F^- in the presence of 100 eq. different anions. (**Figure S3**)..........S4
4. UV-vis absorption spectra of dry **PI-OSi** solutions (10 μM in DMF) after adding F^- and subsequent adding trace water. (**Figure S4**)..........S5
5. Color change of **PI-OSi** solutions (1 mM in DMF) after adding F^- and subsequent adding trace water. (**Figure S5**)..........S6
6. UV-vis absorption spectra of **PI-OSi** solutions (10 mM in 1,4-Dioxane, DMAc, THF) after adding F^- and subsequent adding trace water. (**Figure S6**)..........S7
7. Structure of model compounds (**Figure S7**)..........S8

1. Synthesis and characterization of PI-OH and PI-OSi.

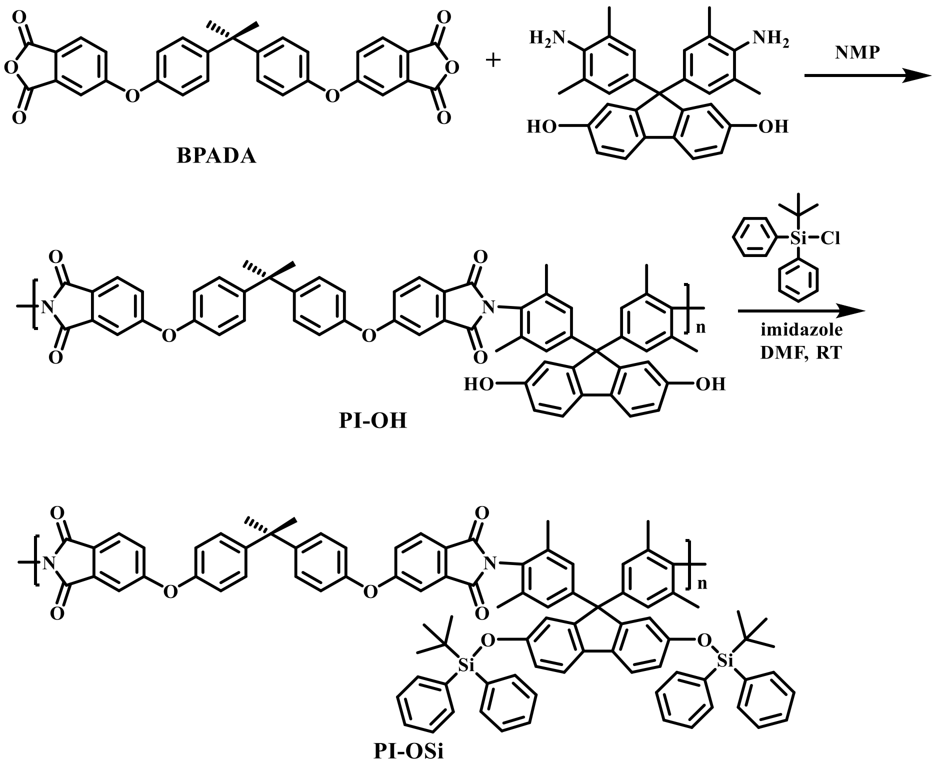

Scheme S1. Synthesis of hydroxyl-containing **PI-OH** and *tert*-butyldiphenylsilyl-containing **PI-OSi**.

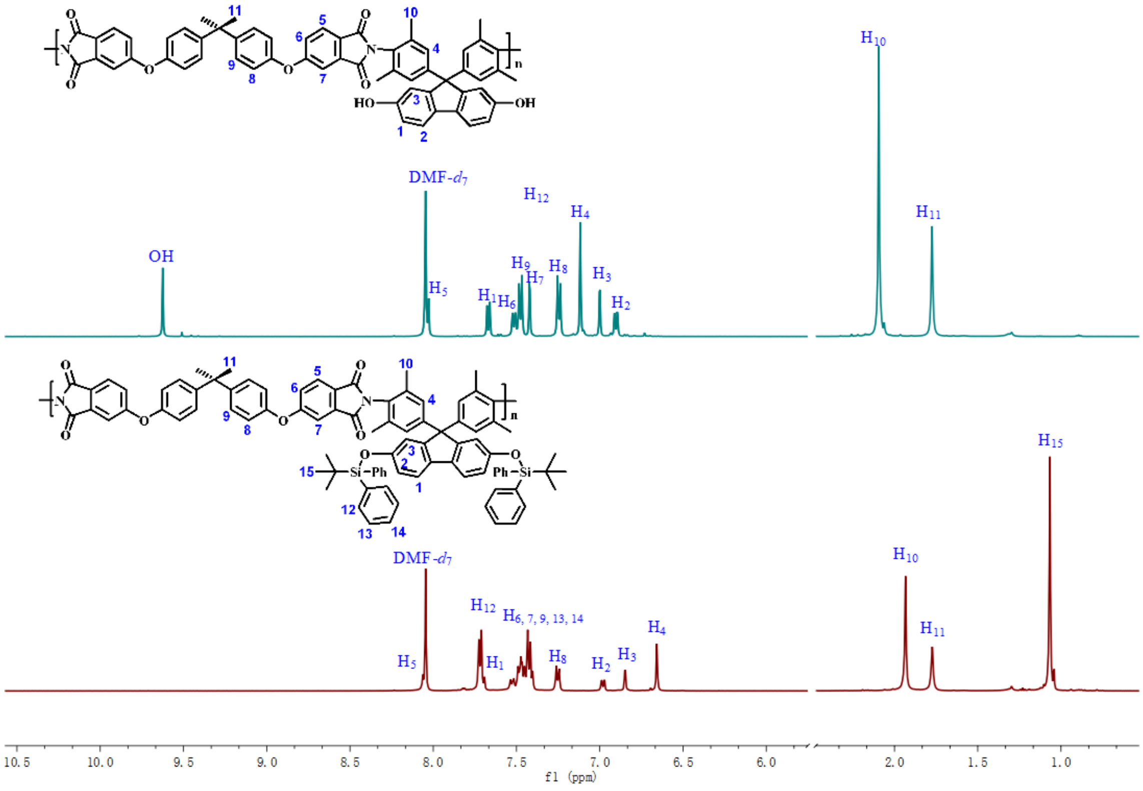

Figure S1. ^{1}H NMR spectra of (a) **PI-OH** and (b) **PI-OSi**.

2. A_{322}/A_{288} value of PI-OSi upon adding 100 eq. F^- and five cations.

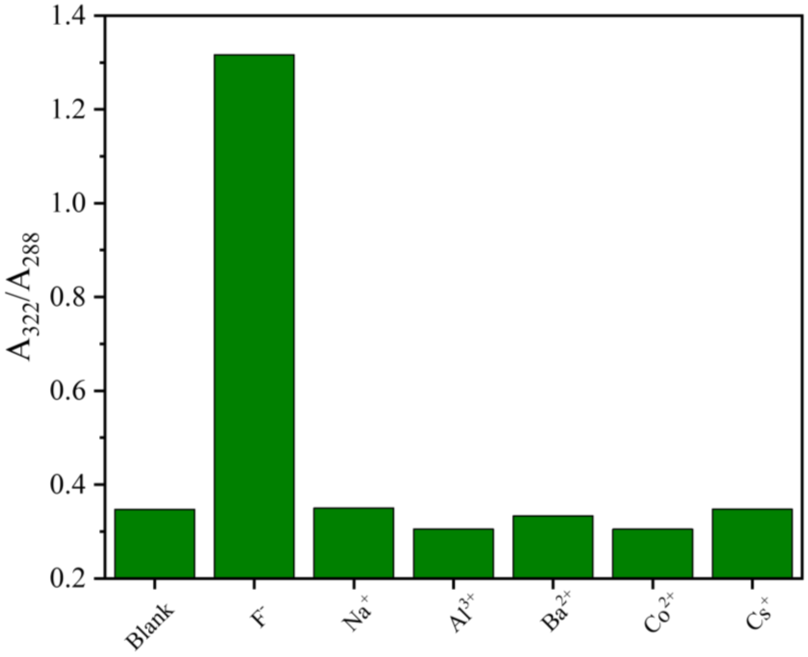

Figure S2. A_{322}/A_{288} value of **PI-OSi** upon adding 100 eq. F^- and five cations.

3. UV-vis absorption spectra of PI-OSi upon the addition of 100 eq. F^- in the presence of 100 eq. different anions.

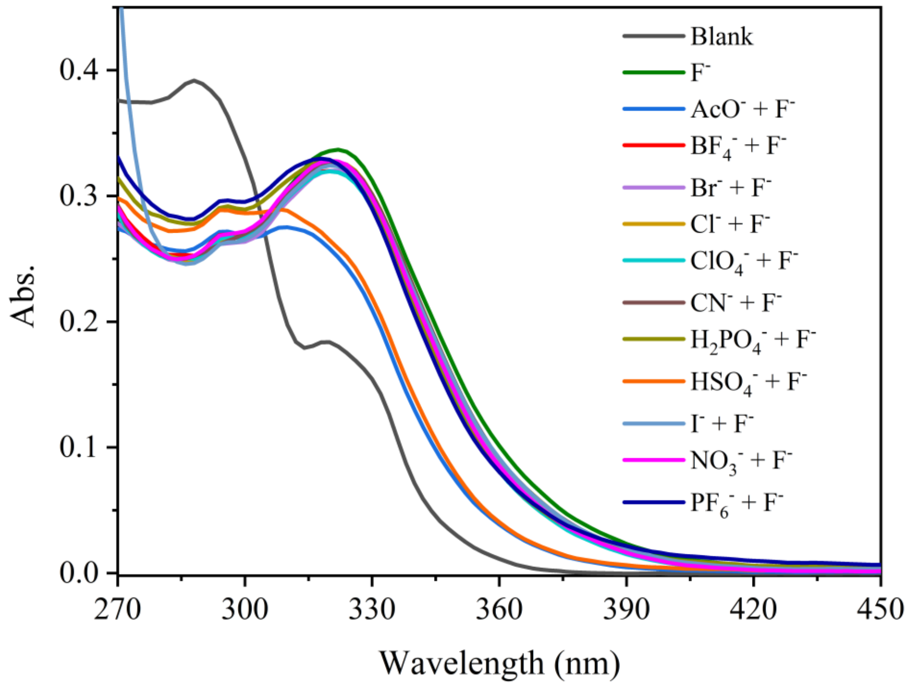

Figure S3. UV-vis absorption spectra of **PI-OSi** upon the addition of 100 eq. F^- in the presence of 100 eq. different anions.

4. UV-vis absorption spectra of dry PI-OSi solutions (10 μM in DMF) after adding F^- and subsequent adding trace water.

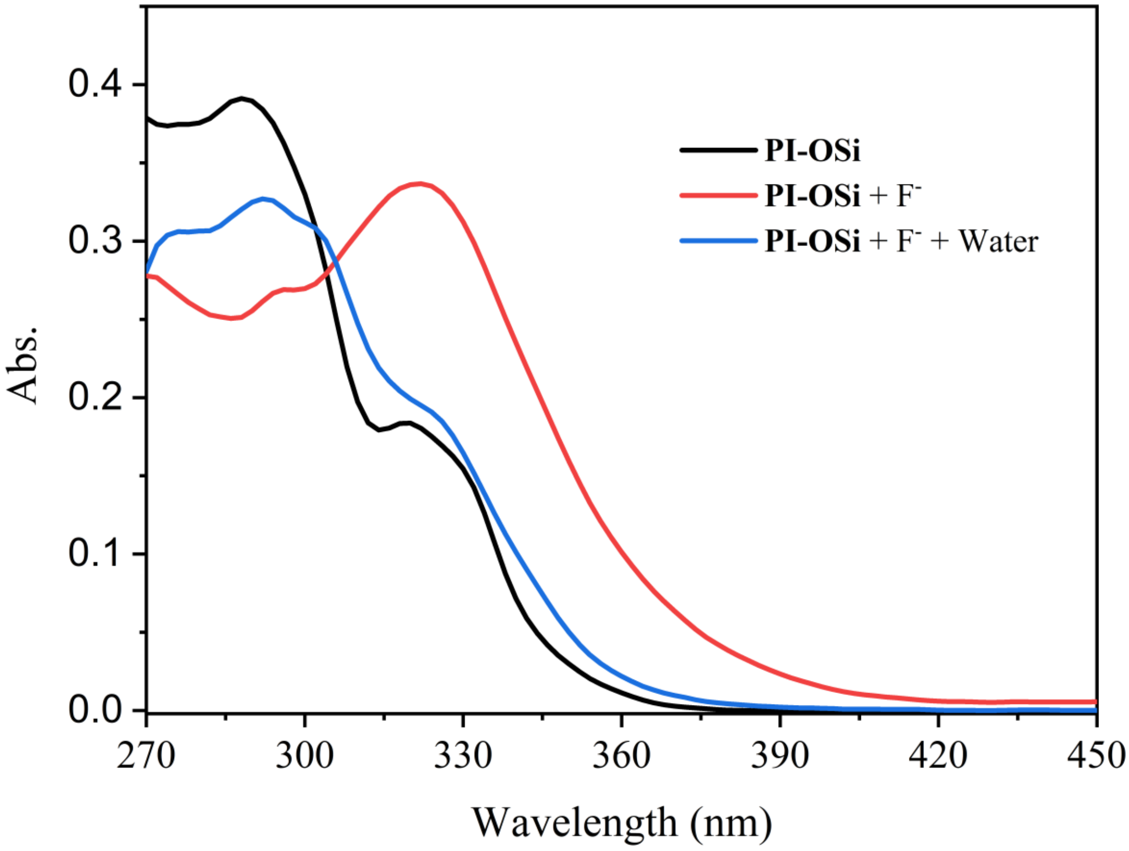

Figure S4. Change in UV-vis absorption spectra for dry **PI-OSi** solutions (10 μM in DMF) after adding F^- and subsequent adding trace water.

5. Color change of PI-OSi solutions (1 mM in DMF) after adding F^- and subsequent adding trace water.

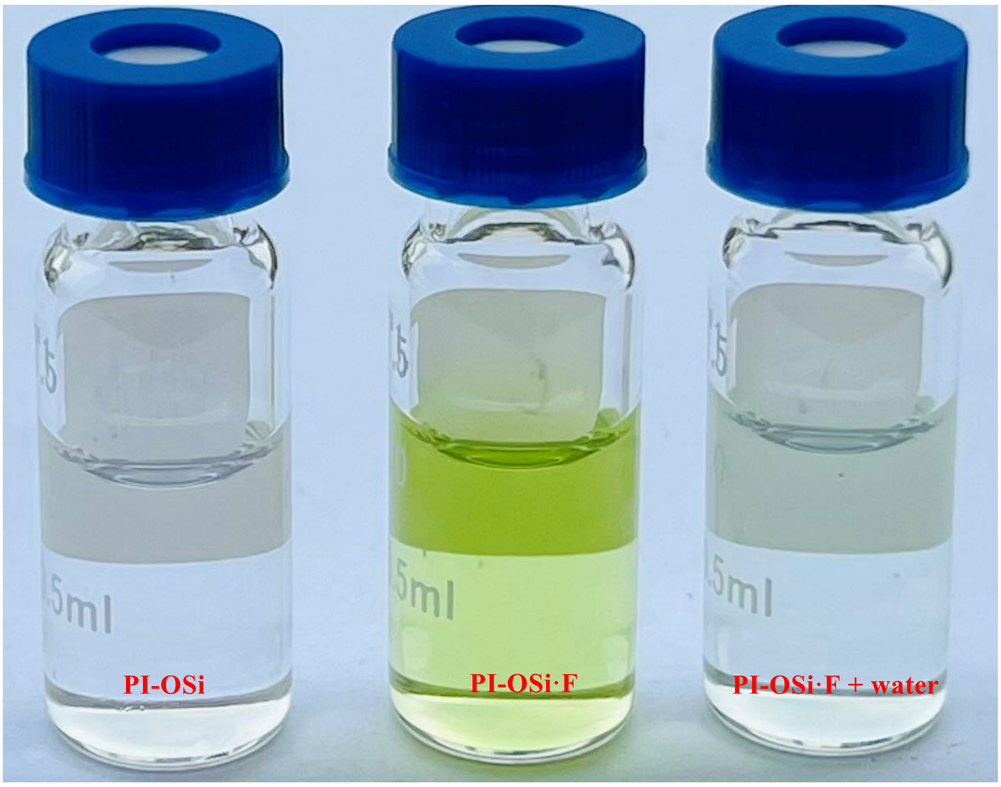

Figure S5. Changes in color of **PI-OSi** solutions (1 mM in DMF) with the addition of F^- (6 eq.) and subsequent addition of trace water (5%, v/v).

6. UV-vis absorption spectra of dry PI-OSi solutions (10 μM in 1,4-Dioxane, THF, DMAc) after adding F^- and subsequent adding trace water.

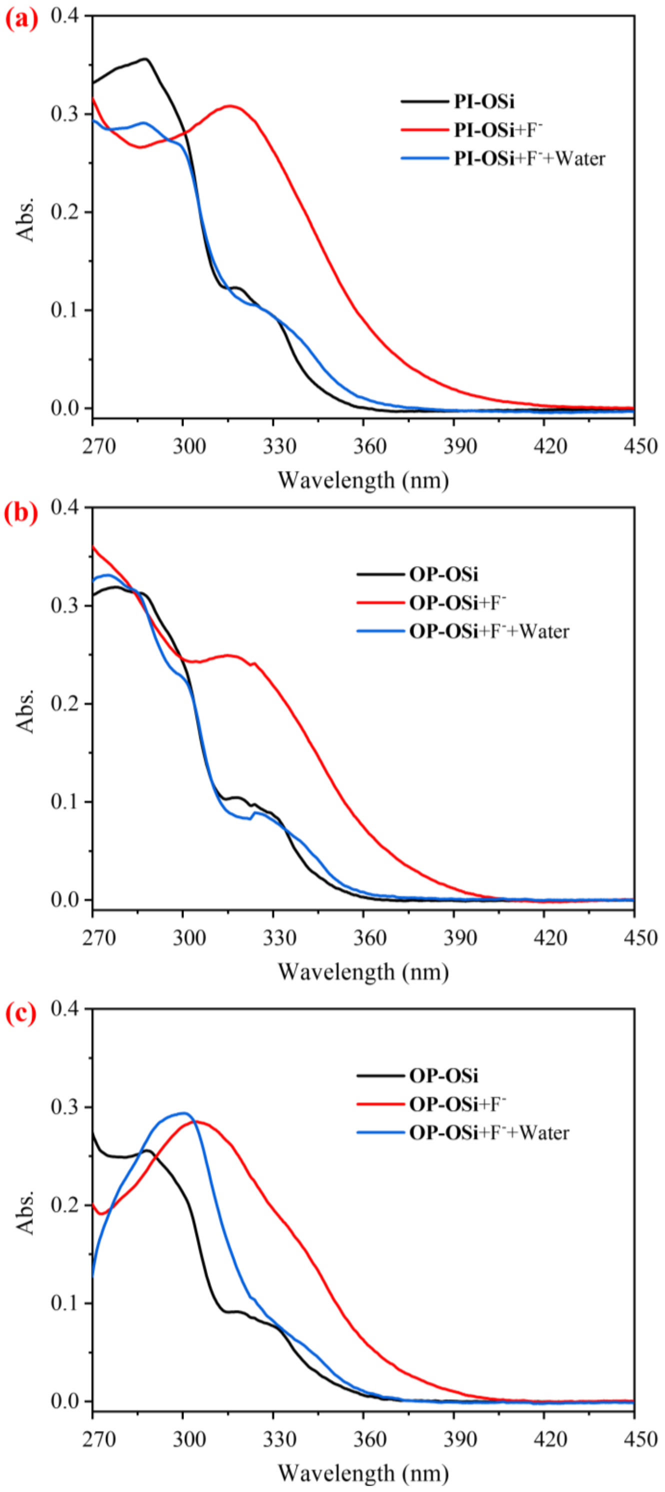

Figure S6. Change in UV-vis absorption spectra for dry **PI-OSi** solutions (10 μM in (a) 1,4-Dioxane, (b) THF, (c) DMAc) after adding F^- and subsequent adding trace water.

7. Structure of model compounds

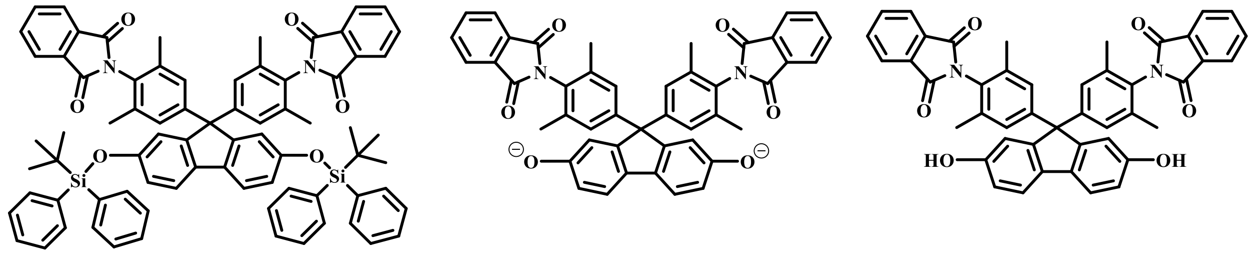

MOSi **MO⁻** **MOH**

Figure S7. Structure of model compounds for theoretical calculation.
